# Structural characteristics and evolutionary trajectories of knowledge recombination in the field of AI-driven drug discovery

**DOI:** 10.1038/s41598-026-50574-3

**Published:** 2026-04-27

**Authors:** Jing Xiao, Tianzhu Li, Yiting Luan, Xue Chen

**Affiliations:** 1 School of Business Administration, Guizhou University of Finance & Economics, Guiyang, China; 2 School of Economics and Management, Guizhou Normal University, Guiyang, China

**Keywords:** Knowledge recombination, AI-driven drug discovery, Patent analysis, Structural characteristics, Evolutionary trajectories, Computational biology and bioinformatics, Drug discovery, Mathematics and computing

## Abstract

The rapid evolution of artificial intelligence (AI) and its profound integration with the pharmaceutical industry essentially constitute a process of knowledge recombination. Yet, empirical evidence systematically characterizing the structural and evolutionary dynamics of this phenomenon in AI-driven drug discovery remains scarce. Utilizing 494 granted invention patents from Chinese AI pharmaceutical firms (2011–2024), this study establishes a multi-method framework—integrating IPC co-occurrence networks, semantic similarity, LDA topic modelling, and longitudinal sliding-window analysis—to systematically characterize this cross-domain recombination. Structurally, the network exhibits a “sparse yet concentrated” topology. Bioinformatics (G16B) emerges as the critical bridging hub, facilitating distant recombination characterized by high combinatorial intensity but low cognitive similarity. Longitudinally, the network’s evolution reveals a “temporal lag” in knowledge integration, marked by declining network density alongside a rising average degree. The technological dominance transitioned progressively from traditional pharmaceuticals (A61K) to computing (G06F), and ultimately to bioinformatics (G16B) and multidisciplinary integration. These findings offer an empirical lens into the micro-level dynamics of interdisciplinary knowledge convergence, providing preliminary insights that may inform capability development and innovation policymaking.

**Supplementary Information:**

The online version contains supplementary material available at 10.1038/s41598-026-50574-3.

## Introduction

In November 2020, DeepMind’s AlphaFold2 achieved a breakthrough in protein structure prediction, marking a watershed moment in the integration of artificial intelligence (AI) and pharmaceutical research^1^. This exemplifies the transformative technological shock that fundamentally reshapes competitive landscapes^2^. The integration of AI into pharmaceutical research and development (R&D) represents more than an emerging business opportunity—it may represent a significant restructuring of pharmaceutical innovation through the recombination of distant knowledge domains.Accordingly, numerous firms dedicated to AI-driven drug discovery have emerged. AI-driven drug discovery encompasses the application of artificial intelligence technologies, including machine learning and deep learning, to drug discovery, design, and development processes for screening, optimising, and predicting drug targets and lead compounds^3,4^.

At the heart of this transformation lies knowledge recombination—the process of combining previously separate knowledge components to create novel solutions^5,6^. Innovation scholars recognise that breakthrough innovations often emerge from recombining knowledge across distant domains rather than from incremental improvements within established fields^7^. However, integrating AI into pharmaceutical R&D presents unique challenges: these domains are characterised by distinct knowledge structures, technological paradigms, and problem-solving approaches^8^. This cognitive distance simultaneously creates opportunities for novel recombination and poses barriers to successful integration^9^.

Despite growing interest, systematic empirical evidence on how knowledge from these distant domains is actually recombined remains notably scarce. Existing studies have examined AI applications in specific pharmaceutical tasks—molecular design^10^, target identification^11^—but primarily through case studies or technical demonstrations rather than large-scale patent analysis. Consequently, two fundamental questions remain empirically unaddressed.

**First (RQ1)**,** what are the structural characteristics of knowledge recombination in AI-driven drug discovery?** Specifically, is recombination activity sparse or dense across the knowledge space? Is it uniformly distributed or concentrated in specific combinations? Which knowledge domains serve as bridging hubs that connect AI technologies and pharmaceutical sciences—two domains characterised by substantial cognitive distance? And what thematic content underlies these structural patterns? **Second (RQ2)**,** how does the knowledge recombination network evolve as the field matures?** Specifically, how do network scale, connectivity, and hub positions change over time? What temporal dynamics characterise the integration of new knowledge elements into the existing network?

We focus on granted invention patents from Chinese AI pharmaceutical firms for three reasons. First, China has emerged as a major hub for AI pharmaceutical innovation, with a rapidly growing number of dedicated firms providing sufficient patent volume for systematic analysis. Second, Chinese AI pharmaceutical firms are predominantly new ventures whose knowledge recombination is not constrained by the path dependence of traditional pharmaceutical paradigms. Unlike the gradual AI integration by established Western pharmaceutical companies, most Chinese AI pharmaceutical firms (e.g., Insilico Medicine, XtalPi) have been AI-centric since inception, exhibiting “native” knowledge recombination patterns and providing a clean sample for observing distant knowledge domain integration. Third, the research context ensures data completeness and accessibility. The IncoPat patent database comprehensively covers Chinese AI pharmaceutical firm patents, and the standardised International Patent Classification (IPC) system facilitates knowledge element identification, yielding 494 granted invention patents from 58 firms spanning 2011–2024—a sample of sufficient size for network analysis.

**This study provides the systematic empirical characterisation of knowledge recombination in AI-driven drug discovery.** We address the gap stemming from weak empirical research by answering two descriptive research questions through multi-method patent analysis.Addressing RQ1, we reveal four sets of structural characteristics. First, knowledge recombination exhibits a “sparse yet concentrated” network architecture: only 14.71% of possible knowledge element pairs co-occur, yet co-occurrence frequencies follow a long-tail distribution with a few core combinations dominating. Second, bioinformatics (G16B) serves as the central bridging hub, displaying the highest degree centrality (connecting 46% of network nodes), betweenness centrality, and closeness centrality—a unique structural position attributable to its dual computational-biomedical nature that enables it to “speak the language” of both AI and pharmaceutical sciences. Third, comparison between combinatorial intensity (Jaccard coefficient) and semantic similarity (cosine similarity) reveals a critical distinction: AI-driven combinations (e.g., G06N-G16B) exhibit high combinatorial intensity despite low semantic similarity, providing direct evidence for distant recombination across cognitive boundaries. Fourth, LDA topic modelling based on a multi-metric evaluation framework identifies eight thematic domains, including two distinct AI-related paradigms: a generative paradigm and a predictive paradigm.Addressing RQ2, sliding-window network analysis documents the evolutionary trajectory of knowledge recombination. Network scale expanded substantially over the observation period: node count increased from 3 to 34, and edge count from 2 to 88. Notably, network density declined (from 0.667 to 0.157) concurrent with rising average degree (from 1.33 to 5.18)—indicating continuous influx of new technologies alongside strengthening integration among core elements. This pattern reflects temporal lag in knowledge integration: newly entering elements dilute overall connectivity while core nodes consolidate their positions. Bioinformatics (G16B) exemplifies this trajectory, evolving from a peripheral position in early windows to the dominant hub in later periods.

Methodologically, by integrating longitudinal sliding-window network analysis with IPC co-occurrence, semantic similarity, and LDA topic modelling, this study proposes a multi-dimensional framework that aims to capture the complex structural and evolutionary facets of cross-domain knowledge recombination more comprehensively than single-method approaches.

Our findings may offer practical insights for multiple stakeholders. AI pharmaceutical firms can leverage the identified core combinations and hub positions to guide capability development—particularly the strategic importance of bioinformatics as a bridging domain. Policymakers can use the documented evolutionary patterns to calibrate support strategies, recognising that new technologies require time to integrate into the existing knowledge network. Educational institutions can align interdisciplinary curricula with the bridging role of bioinformatics in connecting computational and biomedical paradigms.

The remainder of this paper is organised as follows. Section  2 reviews the literature on knowledge recombination and technological convergence, identifying two empirical gaps. Section  3 describes data sources and analytical methods. Section  4 presents structural characteristics of knowledge recombination (addressing RQ1), including network topology, bridging hubs, combinatorial intensity versus semantic similarity, and thematic content. Section  5 presents evolutionary analysis (addressing RQ2), documenting network dynamics through sliding-window analysis. Section  6 summarises findings, discusses implications, acknowledges limitations, and concludes with directions for future research.

## Background and related work

### AI-driven drug discovery: an emerging cross-domain field

AI-driven drug discovery has emerged as a rapidly growing field that applies artificial intelligence technologies—including machine learning, deep learning, and neural networks—to pharmaceutical research and development processes such as target identification, molecular design, and lead compound optimisation^3,11^. The landmark achievement of AlphaFold2 in protein structure prediction^1^ exemplified the transformative potential of this integration, and subsequent advances such as AlphaFold3 have further extended AI capabilities to biomolecular interaction prediction^12^.

This field represents a convergence of artificial intelligence and pharmaceutical sciences—two domains characterised by distinct knowledge structures, technological paradigms, and problem-solving approaches^8^. AI technologies are grounded in data-driven modelling and computational algorithms, whereas pharmaceutical sciences rely on experimental validation and biological assays. The cognitive distance between these domains creates both opportunities for novel innovation and challenges for successful integration^9^. Innovation scholars have long recognised that combining knowledge from distant domains can generate disproportionate impact^7,13^, yet such integration requires bridging mechanisms that span the gap between different epistemic cultures.

Existing research on AI-driven drug discovery has primarily examined AI’s technical performance in specific pharmaceutical tasks. Studies have demonstrated impressive capabilities in molecular design through deep neural networks^14^(Ma et al. 2015), de novo compound generation through reinforcement learning^15^, and functional genomics applications^16^. Comprehensive reviews have summarised these multifaceted advances and envisioned AI’s transformative potential^17,3^. However, these studies focus on what AI can accomplish technically, rather than on how knowledge from AI and pharmaceutical domains is actually recombined. Systematic empirical evidence on the structural characteristics of knowledge recombination in this cross-domain field remains notably scarce.

### Patent analysis for characterising technological fields

Patent documents provide a systematic record of technological innovation, offering unique advantages for analysing knowledge recombination patterns. Each patent must classify its technical content using standardised systems such as the International Patent Classification (IPC), and when a patent is assigned multiple IPC codes, this indicates that knowledge from different technological domains has been recombined^18^. By analysing which IPC codes co-occur within patents, researchers can reveal knowledge recombination patterns and identify which domains frequently combine to generate innovations.

Co-occurrence network analysis has proven valuable for understanding knowledge structures in various technological domains. Previous studies demonstrated this approach by analysing millions of US patents, revealing that technological combination follows power-law distributions with most inventions involving rare, atypical combinations^18^.Network centrality measures—including degree centrality, betweenness centrality, and closeness centrality—provide tools for identifying structurally important knowledge elements within co-occurrence networks^19,20^. Recent patent studies have applied these methods to examine technological evolution in domains such as 5G technology^21,22^ and generative AI^23^.

To capture thematic content beyond formal IPC classifications, researchers have increasingly employed topic modelling techniques. Latent Dirichlet Allocation (LDA) extracts latent semantic themes from patent texts, revealing what technological combinations aim to achieve^24^. Studies have applied LDA to identify technology trends in healthcare AI^25^ and to forecast emerging technological opportunities^26^. Combining IPC-based structural analysis with topic modelling provides complementary perspectives: the former reveals which knowledge domains combine, while the latter uncovers the thematic content underlying these combinations.

For investigating evolutionary dynamics, sliding-window network analysis offers an effective approach. By constructing sequential networks across overlapping time windows, researchers can trace how network properties—including scale, connectivity, and centrality distributions—change over time. This method has been applied to track technological evolution in various domains^27^, revealing patterns of network expansion, consolidation, and hub emergence. However, existing patent-based studies typically provide cross-sectional snapshots aggregated across extended periods^18^, rather than tracking how recombination networks evolve within specific emerging fields.

### Research gaps and questions

Based on the foregoing review, this study addresses two empirical gaps.

**Gap 1**: The structural characteristics of knowledge recombination in AI-driven drug discovery remain empirically uncharacterised. Existing research has examined AI’s technical capabilities in various drug discovery tasks, yet systematic empirical evidence on the structural characteristics of knowledge recombination in this cross-domain field is lacking. This study asks: **What are the structural characteristics of knowledge recombination in AI-driven drug discovery (RQ1)?**

**Gap 2**: The evolutionary trajectory of knowledge recombination networks in this emerging field lacks longitudinal documentation. This study asks: **What is the evolutionary trajectory of the knowledge recombination network in AI-driven drug discovery as the field matures (RQ2)?**

By addressing these two gaps, this study provides a descriptive empirical characterisation of knowledge recombination in AI-driven drug discovery.

## Data and methods

This study defines AI pharmaceutical firms as enterprises employing artificial intelligence as a core driving force, either specialising in drug R&D or deeply integrating AI technologies throughout drug discovery and development processes. Using granted invention patents from these firms as the research sample, this section describes the research methods and data sources.

### Research methods

To address RQ1, five analytical methods were employed. IPC co-occurrence network construction followed established approaches^18^, extracting the first four digits of IPC codes and constructing a raw co-occurrence frequency matrix. The choice of the four-digit IPC subclass as the analytical unit warrants justification. IPC codes follow a hierarchical structure: section, class, subclass, main group, and subgroup. In co-occurrence-based studies of knowledge recombination and technological relatedness, the subclass level has been widely adopted as the standard analytical unit. Knowledge-relatedness measures have been constructed using patent co-classifications mapped to technological fields derived from IPC subclasses, establishing this granularity as appropriate for capturing the knowledge base underlying firms’ technological diversification^28^. Combinatorial invention patterns across US patents have similarly been analysed at a comparable taxonomic level^29^. Moreover, co-classification analysis using 138,751 PCT patents at both three- and four-digit IPC levels has shown that classification linkages are already weak at the four-digit level, suggesting that finer-grained disaggregation would yield increasingly sparse and fragmented network structures^30^.This analytical granularity continues to be widely adopted in recent studies across diverse research contexts. Technological convergence has been operationalised as the co-classification of patents into multiple four-digit IPC codes to identify emergent topics^31^, and overlay basemaps for USPTO patent data have been developed at both the three- and four-digit IPC levels to analyse technological distance and portfolio diversity^32^. More recently, convergence distance has been measured based on IPC-level differences in patent backward and forward citations to predict promising technologies^33^, and a Knowledge Origin Re-Combination Index (KORCI) has been constructed by tracking the recombination of technological components listed in patent classifications across artificial intelligence, computer technology, and pharmaceutical sectors^34^. In studies of knowledge recombination at the inventor level, the first four digits of the IPC classification number have been explicitly employed to represent knowledge elements for measuring the diversity and novelty of recombinant activities^35^. These studies collectively confirm that the four-digit IPC subclass remains the standard analytical unit for co-occurrence-based studies of knowledge recombination and technological convergence.To empirically verify this expectation, we conducted a supplementary analysis using six-digit (main group) IPC codes. The finer-grained classification expanded the number of knowledge elements from 37 to 184, but the matrix fill rate dropped from 14.71% to 8.80%, with the majority of non-zero pairs co-occurring in only one or two patents. Network fragmentation was also evident, with 28.3% of nodes exhibiting a degree of five or fewer. These results confirm that the four-digit subclass level provides the appropriate balance between analytical granularity and network reliability for a sample of this size (494 patents). The complete six-digit co-occurrence matrix is reported in Appendix C.The Jaccard similarity coefficient was used to normalise co-occurrence frequencies into combinatorial intensity measures. Semantic similarity between knowledge domains was computed using the BGE-large-zh-v1.5 embedding model, with cosine similarity calculated between vector representations of IPC textual descriptions. Network centrality analysis employed degree centrality, PageRank, betweenness centrality, and closeness centrality to identify influential nodes and bridging hubs^19,20^. Latent Dirichlet Allocation (LDA) topic modelling was applied to patent titles and abstracts to uncover thematic content underlying structural patterns^24^. Detailed procedures for each method are presented in Structural characteristics Section .

To address RQ2, a sliding-window approach was employed to trace the evolutionary trajectory of the knowledge recombination network. The 2011–2024 observation period was divided into ten overlapping five-year windows (2011–2015, 2012–2016, …, 2020–2024) with one-year increments. For each window, a Jaccard-weighted co-occurrence network was constructed, and the following indicators were computed: node count (number of knowledge elements), edge count (number of co-occurrence relationships), network density (ratio of actual to possible edges), average degree (mean connections per node), and clustering coefficient (local clustering tendency). Centrality measures were also tracked across windows to identify shifts in hub positions. Network construction and analysis were performed using Python (NetworkX, Pandas) with Spring Layout (Fruchterman-Reingold) algorithm for visualisation.

### Data sources

As an emerging field at the intersection of artificial intelligence and traditional pharmaceutical industries, AI-driven drug discovery lacks unified official standards for defining and identifying relevant firms. Currently, tracking developments in this field relies primarily on specialised technology media and industry research institutions. Intellectual Medicine Bureau, a prominent media platform in China focusing on AI-driven drug discovery, has long tracked the development and application of frontier technologies in biotechnology domains, including AI drug development and synthetic biology, maintaining high industry recognition and information accuracy.

**Fig. 1:**
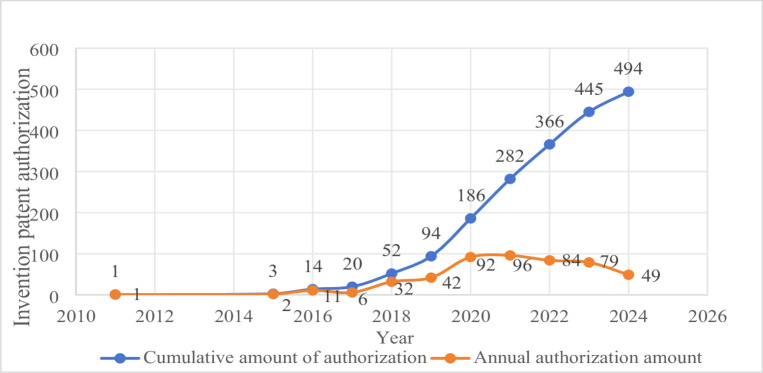
Trends in granted invention patent volumes.

Based on the AI pharmaceutical industry report released by Intellectual Medicine Bureau in 2023, this study initially obtained a list of 92 Chinese AI pharmaceutical firms. To ensure data accuracy and representativeness, we verified the primary business scope of each firm through business registration information retrieval, excluding firms whose business activities deviated from the core domain of AI-driven drug discovery. Subsequently, we employed the IncoPat database to retrieve granted invention patent information for the remaining firms from 2011 to 2024. Ultimately, 58 firms were identified as holding relevant patents, yielding 494 granted invention patents as the analytical sample. Figure 1 presents the annual and cumulative volumes of granted invention patents. Annual patent grants remained below 11 per year during 2011–2017, increased substantially from 2018 onward (reaching 32 in 2018, exceeding the cumulative total of the preceding seven years), peaked at 96 in 2021, and moderated during 2022–2024.

Two additional data sources were utilised. First, International Patent Classification (IPC) codes were extracted from each patent to serve as knowledge elements for co-occurrence network construction. Second, official textual descriptions of IPC subclasses were obtained from the China National Intellectual Property Administration (CNIPA) to enable semantic similarity computation between knowledge domains.

### Integrated analytical framework

**Fig. 2:**
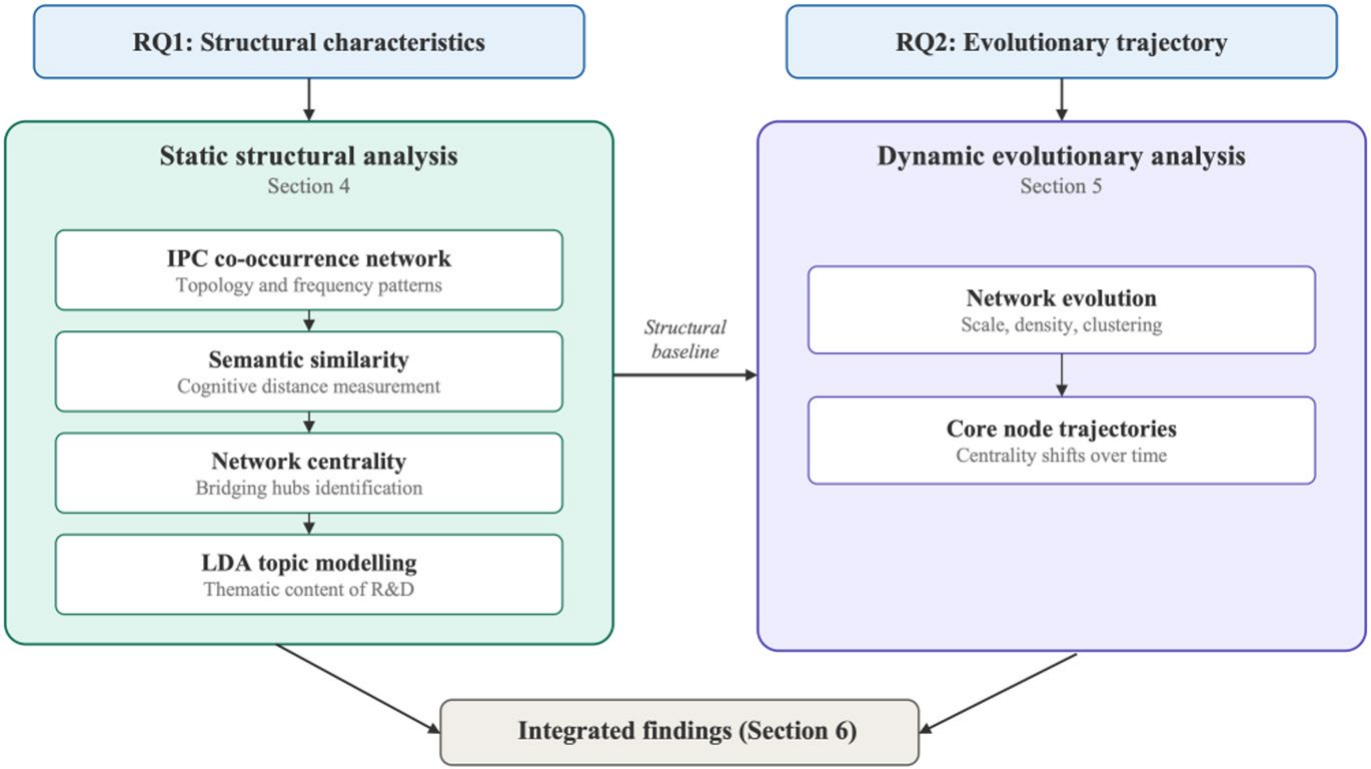
Integrated analytical framework.

To provide a clear roadmap for the overall research design, this subsection articulates the integrated logic connecting the two analytical modules employed in this study. Figure 2 presents the analytical framework.

The static structural analysis (Structural Characteristics Section) addresses RQ1 by characterising the cross-sectional architecture of knowledge recombination across the full observation period (2011–2024). This module proceeds through four sequential sub-analyses: (1) IPC co-occurrence network construction reveals the macro-level topology and frequency patterns of knowledge element combinations; (2) semantic similarity analysis distinguishes combinatorial intensity from cognitive distance, identifying whether high-frequency combinations reflect proximate or distant recombination; (3) network centrality analysis identifies which knowledge elements occupy structurally critical positions as bridging hubs; and (4) LDA topic modelling uncovers the thematic content of R&D activities underlying these structural patterns. Together, these four sub-analyses establish the architectural conditions for cross-domain knowledge recombination.

The dynamic evolutionary analysis (Evolutionary Analysis Section) addresses RQ2 by tracing the evolution of the above structural features over time using sliding-window network analysis. This module consists of two sub-analyses: (1)Tracking the evolution of network-level indicators (node count, edge count, density, average degree, and clustering coefficient) across ten overlapping five-year windows, to document the evolutionary patterns exhibited by network topological indicators over time; and (2)Tracing the changing trajectories of centrality for individual knowledge elements across windows to identify shifts in technological dominance and bridging roles.This module reveals the processual mechanisms underlying the evolution of the knowledge recombination structure — clarifying how the network expands and how core hub positions shift over time.

The two modules are conceptually linked through a baseline–trajectory logic: the static analysis provides the structural baseline by identifying core nodes, dominant combinations, and topological features across the aggregated period, while the dynamic analysis examines whether these baseline features are temporally stable or undergo systematic transformation. For instance, the static analysis identifies bioinformatics (G16B) as the central bridging hub (Network centrality analysis Section); the dynamic analysis then reveals that this hub position was not present from the outset but emerged progressively through a specific evolutionary trajectory (Evolutionary trajectories of core knowledge elements Section). This complementary design enables the study to move beyond a single cross-sectional snapshot and capture both the “architecture” and the “process” of cross-domain knowledge recombination.

## Structural characteristics

This section addresses RQ1 by examining the structural characteristics of knowledge recombination in AI-driven drug discovery. The analysis comprises four subsections.

 Construction of the raw co-occurrence frequency matrix Section constructs a raw co-occurrence frequency matrix based on International Patent Classification (IPC) codes, revealing macro-level distributional characteristics of knowledge recombination, including network sparsity and the long-tail distribution of co-occurrence frequencies. Jaccard similarity matrix and combinatorial intensity–cognitive distance comparison Section builds upon this foundation by constructing a Jaccard similarity matrix to quantify combinatorial intensity, and further compares combinatorial intensity with semantic similarity to distinguish whether high-frequency knowledge combinations reflect cognitive proximity between knowledge domains or represent genuine distant recombination across cognitive boundaries. Network centrality analysis Section conducts network centrality analysis using multi-dimensional centrality measures to identify knowledge domains occupying critical positions within the co-occurrence network. LDA topic modelling Section employs Latent Dirichlet Allocation (LDA) topic modelling to uncover the thematic content of AI-driven drug discovery, complementing the preceding IPC-based analysis—while IPC co-occurrence analysis reveals which knowledge domains combine, topic modelling further elucidates the R&D objectives and application orientations underlying these combinations.

### Construction of the raw co-occurrence frequency matrix

We exported IPC codes from the 494 retrieved granted invention patents. Following established approaches in knowledge recombination research^28,29^,we treated the first four digits of International Patent Classification codes as knowledge elements and constructed a co-occurrence matrix to conduct preliminary analysis of knowledge recombination characteristics in AI pharmaceutical firm patents.Using Python, we extracted the first four digits of IPC codes and removed duplicates within each patent, yielding 37 unique knowledge elements. On this basis, we generated a symmetric raw co-occurrence frequency matrix (see Appendix A). This matrix has dimensions of 37 × 37, where each cell value represents the number of times the knowledge element in that row co-occurs with the knowledge element in that column within the same patent. Diagonal values indicate the number of patents in which each knowledge element appears.

Table 1 presents the high-frequency knowledge elements in granted invention patents in AI-driven drug discovery. These knowledge elements and their combination patterns constitute the core knowledge system underlying AI pharmaceutical firm patents, reflecting knowledge structure characteristics of current AI-driven drug discovery technologies. We mapped IPC codes to corresponding knowledge domains based on the ISI-OST-INPI classification concordance table. Since the latest version of the ISI-OST-INPI concordance table was updated in 2008, and G16 was introduced as a new patent classification after 2019 for “information and communication technology specially adapted for specific application fields”, we mapped G16B to “bioinformatics” and G16C to “cheminformatics” according to the International Patent Classification (Version 2025.01) published by the China National Intellectual Property Administration. The most frequently appearing knowledge element in AI pharmaceutical firm patents is A61K, appearing in 159 of the 494 patents.

**Table 1:** High-frequency knowledge elements in AI pharmaceutical firm patents (Top 10).

IPC Code	ISI-OST-INPI Classification	IPC Description	Count
A61K	Pharmaceuticals	Preparations for medical, dental, or toilet purposes	159
A61P	Pharmaceuticals	Therapeutic activity of chemical compounds or medicinal preparations	145
G16C	–	Computational chemistry; cheminformatics	118
G16B	–	Bioinformatics	107
C07D	Organic fine chemicals	Heterocyclic compounds	87
G06F	Computer technology	Electric digital data processing	71
G06N	Computer technology	Computer systems based on specific computational models	63
C12N	Biotechnology	Microorganisms or enzymes; compositions thereof	43
G01N	Computer technology	Investigating or analysing materials	25
C12Q	Biotechnology	Measuring or testing processes involving enzymes or nucleic acids	23

**Table 2:** presents the top ten knowledge element pairs ranked by raw co-occurrence frequency. To illustrate the technological substance underlying these co-occurrence patterns, we briefly characterise the nature of the recombination reflected in each pair.

Rank	Knowledge Element Pair	Co-occurrence Frequency	Domain Classification
1	A61K–A61P	144	Pharmaceutical preparations – Therapeutic activity
2	A61K–C07D	68	Pharmaceutical preparations – Organic fine chemicals
3	A61P–C07D	62	Therapeutic activity – Organic fine chemicals
4	G06N–G16C	28	Computer technology – Cheminformatics
5	G06N–G16B	28	Computer technology – Bioinformatics
6	G06F–G16C	24	Computer technology – Cheminformatics
7	A61K–C12N	23	Pharmaceutical preparations – Biotechnology
8	A61P–C12N	22	Therapeutic activity – Biotechnology
9	G06F–G16B	16	Computer technology – Bioinformatics
10	G16B–G16C	15	Bioinformatics – Cheminformatics

Table 2. **Top 10 knowledge element pairs by raw co-occurrence frequency**.

The most frequent pair, A61K–A61P (144 co-occurrences), links preparations for medical, dental, or toilet purposes (A61K) with the specific therapeutic activity of chemical compounds or medicinal preparations (A61P), representing the foundational connection between drug formulation and therapeutic evaluation—patents in which novel pharmaceutical preparations are developed and assessed for their efficacy against specific diseases.The second and third pairs, A61K–C07D (68) and A61P–C07D (62), extend this linkage to organic heterocyclic chemistry, reflecting the design and synthesis of heterocyclic compounds as active pharmaceutical ingredients. Representative patents include benzimidazole derivatives developed for hepatitis B treatment (e.g., CN114099517B) and ionisable lipid compounds engineered for nucleic acid delivery (e.g., TWI828461B). Together, these three pairs account for 274 of the total 697 co-occurrences (39.3%), confirming that medicinal chemistry remains the foundational knowledge anchor in AI pharmaceutical firm patents.

The fourth through sixth pairs—G06N–G16C (28), G06N–G16B (28), and G06F–G16C (24)—connect AI and computing technologies with domain-specific informatics. G06N (computational models, including neural networks and machine learning) combined with G16B (bioinformatics) produces technologies such as transformer-based protein structure prediction from single amino acid sequences (e.g., CN115458039B). G06N combined with G16C (cheminformatics) yields applications such as deep neural network-based compound function prediction through gene expression profiling (e.g., CN113178234B). G06F (digital data processing) combined with G16C generates tools such as molecular three-dimensional similarity scoring systems for virtual drug screening (e.g., CN113593655B). These pairs collectively represent the computational core of AI-driven drug discovery, where AI algorithms are integrated with domain-specific informatics to create specialised pharmaceutical tools.

The seventh and eighth pairs, A61K–C12N (23) and A61P–C12N (22), connect pharmaceutical applications with biotechnology, reflecting patents that employ microorganisms, enzymes, or genetic engineering techniques in drug development. The ninth pair, G06F–G16B (16), links data processing with bioinformatics, encompassing technologies such as gene alignment acceleration hardware and biomedical knowledge graph construction (e.g., CN111782818B). The tenth pair, G16B–G16C (15), bridges bioinformatics and cheminformatics, represented by patents that integrate genomic data with chemical structure analysis, such as drug–target toxicity prediction models built on CRISPR gene perturbation data (e.g., CN113380341B).

Preliminary statistical analysis, as shown in Table 3, revealed the following findings:

**First**,** associations between knowledge elements exhibited sparse distribution characteristics.** The raw co-occurrence frequency matrix contains 1,369 (37 × 37) cells, with a theoretical maximum of 666 [(1,369 − 37) / 2] possible pairwise co-occurrences between different knowledge elements. Only 98 pairs of different knowledge elements actually co-occurred in the matrix, accounting for 14.71% of the theoretical maximum. This indicates that most knowledge elements lack direct associative relationships, and the raw co-occurrence frequency matrix exhibits typical sparse matrix characteristics.

**Second**,** the distribution of co-occurrence frequencies was highly uneven.** The total co-occurrence frequency was 697, with a maximum of 144, a minimum of 1, a mean of 7.11, and a median of 2. These data indicate that among co-occurring knowledge element pairs, a small number exhibited high-frequency co-occurrence patterns whilst the majority showed low-frequency co-occurrence.

**Fig. 3:**
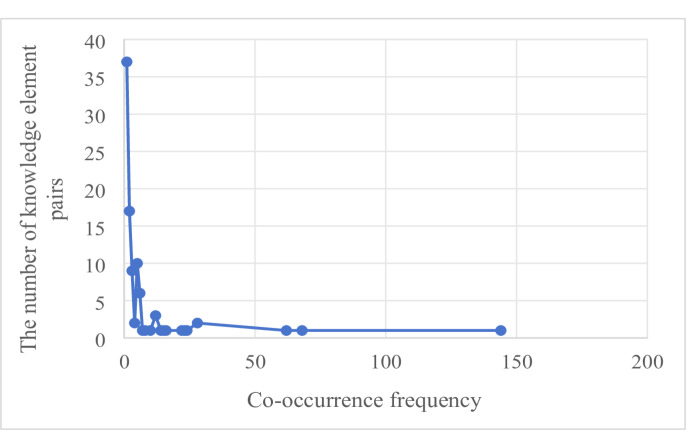
Co-occurrence frequency and knowledge element pairs quantity.

**Third**,** knowledge element co-occurrence frequencies exhibited a typical long-tail distribution.** The high-frequency co-occurrence of a small number of knowledge element pairs significantly elevated the mean co-occurrence frequency, as evidenced by the mean (7.11) being approximately 3.56 times the median (2). As illustrated in Fig. 3, the line chart plotting co-occurrence frequency against the number of knowledge element pairs displays a pronounced “long-tail” pattern, further validating the distributional characteristic wherein a few core knowledge element pairs co-occur at high frequency whilst most pairs co-occur at low frequency.

**Table 3:** Preliminary statistical analysis of the raw co-occurrence frequency matrix.

Indicator	Value
Co-occurring knowledge element pairs	98 pairs
Total co-occurrence frequency	697
Maximum co-occurrence frequency	144
Minimum co-occurrence frequency	1
Mean co-occurrence frequency	7.11
Median co-occurrence frequency	2

### Jaccard similarity matrix and combinatorial intensity–cognitive distance comparison

To facilitate comparison and support subsequent network analysis, we normalised the raw co-occurrence frequency matrix using the Jaccard similarity coefficient:

$$Jaccard\left( {A,{\text{ }}B} \right){\text{ }} = {\text{ }}A{\text{ }} \cap {\text{ }}B{\text{ }}/{\text{ }}A{\text{ }} \cup {\text{ }}B$$

where A and B denote knowledge elements, A ∩ B represents the number of patents containing both elements, and A ∪ B represents the number of patents containing either element minus the overlap. The Jaccard coefficient measures combinatorial intensity—the proportion of patents in which two knowledge elements co-occur relative to all patents containing either element. Values range from 0 to 1, with higher values indicating stronger combinatorial associations between knowledge elements. The complete Jaccard similarity matrix is provided in Appendix B.

While the Jaccard coefficient effectively quantifies combinatorial intensity at the behavioural level, high-frequency co-occurrence alone does not indicate whether the combined domains are cognitively proximate or distant. To distinguish combinatorial intensity from cognitive distance, we computed the semantic similarity between knowledge domains using the BGE-large-zh-v1.5 pre-trained embedding model (BAAI/bge-large-zh-v1.5). Official textual descriptions of each IPC subclass were obtained from the China National Intellectual Property Administration and encoded into fixed-dimensional sentence vectors with normalised embeddings. Cosine similarity was then calculated between vector pairs, where higher values indicate greater semantic proximity between knowledge domains.

Table 4 presents the top ten knowledge element pairs ranked by Jaccard coefficient, together with their cosine similarity values. This juxtaposition enables comparison between combinatorial intensity (how frequently domains are combined in practice) and cognitive distance (how semantically close the domains are in their technical definitions).

**Table 4:** Top 10 combinatorial intensity knowledge element pairs with their cognitive distance.

Knowledge Element Pair	Cosine Similarity	Jaccard Coefficient	Domain Classification
A61K-A61P	0.6853	0.9	Pharmaceutical preparations – Therapeutic activity
A61K-C07D	0.7398	0.38	Pharmaceutical preparations – Organic fine chemicals
A61P-C07D	0.643	0.36	Therapeutic activity – Organic fine chemicals
G06N-G16B	0.3775	0.2	Computer technology – Bioinformatics
G06N-G16C	0.509	0.18	Computer technology – Cheminformatics
G06F-G16C	0.4205	0.15	Computer technology – Cheminformatics
A61K-C12N	0.7888	0.13	Pharmaceutical preparations – Biotechnology
A61P-C12N	0.633	0.13	Therapeutic activity – Biotechnology
G06F-G16B	0.4484	0.1	Computer technology – Bioinformatics
G16B-G16C	0.5034	0.07	Bioinformatics – Cheminformatics

Two findings emerged from this analysis.

First, AI pharmaceutical firms have not departed from traditional pharmaceutical foundations. The three highest-ranking pairs by combinatorial intensity are all within or closely adjacent to the pharmaceutical domain: A61K-A61P (Jaccard = 0.90, cosine = 0.6853), A61K-C07D (Jaccard = 0.38, cosine = 0.7398), and A61P-C07D (Jaccard = 0.36, cosine = 0.6430). These pairs exhibit both high combinatorial intensity and high semantic similarity, indicating cognitively proximate recombination among knowledge domains that share similar technical vocabularies and research paradigms. Pharmaceutical preparation (A61K), therapeutic activity (A61P), and organic fine chemistry (C07D) remain the foundational knowledge anchors in AI-driven drug discovery.

Second, AI-driven combinations exhibit high combinatorial intensity despite low semantic similarity, providing direct evidence for distant recombination across cognitive boundaries. The AI-informatics pairs—G06N-G16B (Jaccard = 0.20, cosine = 0.3775), G06N-G16C (Jaccard = 0.18, cosine = 0.5090), G06F-G16C (Jaccard = 0.15, cosine = 0.4205), and G06F-G16B (Jaccard = 0.10, cosine = 0.4484)—display a pattern of knowledge combinations. Their Jaccard coefficients indicate substantial combinatorial activity, yet their cosine similarity values are considerably lower than those of the pharmaceutical pairs. This contrast demonstrates that AI technologies do not function in isolation but integrate deeply with domain-specific informatics—bioinformatics and cheminformatics—to form specialised solutions.

### Network centrality analysis

In this section, we imported the Jaccard similarity matrix into Gephi software and employed multi-dimensional measures to conduct network centrality analysis, identifying core nodes within the knowledge system. Specifically, core knowledge elements are defined as those possessing both influence (measured through degree centrality and PageRank) and brokerage capacity (measured through betweenness centrality). Knowledge elements simultaneously exhibiting influence and brokerage capacity occupy strategic positions within the co-occurrence network.

**Fig. 4:**
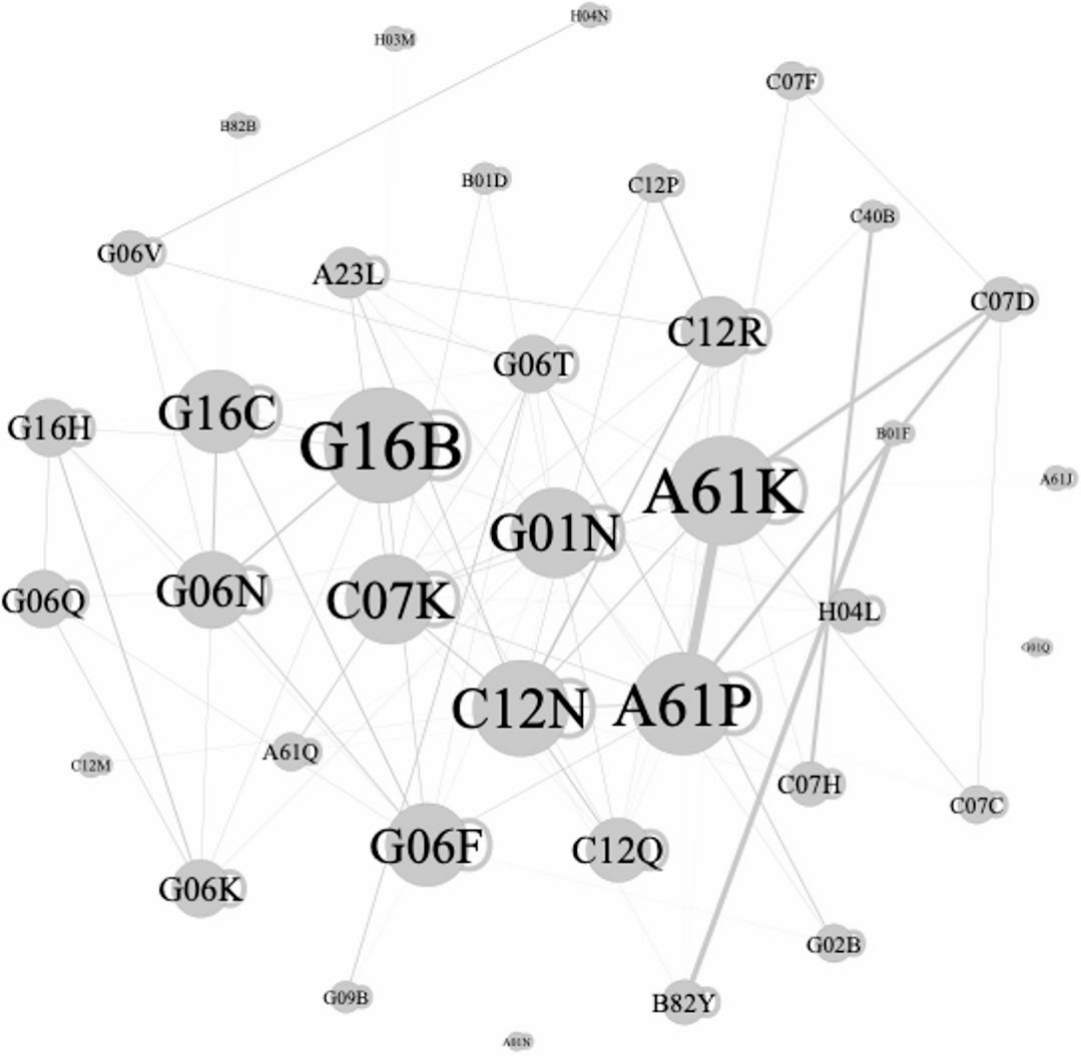
Knowledge element influence network diagram.

1. **Identification of knowledge element influence.** Degree centrality measures the number of connections a node possesses, reflecting direct connections of knowledge elements and embodying their direct influence. However, degree centrality is a local indicator, as a knowledge element’s degree centrality is determined entirely by the number of knowledge elements directly connected to it. Therefore, we introduced PageRank as a complementary indicator to measure knowledge element influence. PageRank considers not only the quantity of connections but also their quality—nodes connected to important nodes are themselves more important. Through an iterative algorithm, PageRank recursively distributes influence across all knowledge elements in the network. PageRank accounts for global network structure; a node’s PageRank value is indirectly influenced by all nodes in the network, meaning two nodes may influence each other through multiple paths even without direct connections. The influence rankings derived from degree centrality and PageRank were nearly identical; thus, this study used degree centrality to construct the knowledge element influence network diagram (Fig. 4). Knowledge elements with high influence closely corresponded to knowledge element pairs with high co-occurrence intensity, indicating that the integration of high co-occurrence intensity knowledge element pairs exerts significant influence on technological development in AI-driven drug discovery. For example, the integration between G06N (representing AI technologies) and G16B (representing bioinformatics) carries substantial significance for technological advancement in AI-driven drug discovery.

As shown in Table 5, G16B (bioinformatics) ranked first in both degree centrality and PageRank. A degree centrality value of 17 indicates that G16B co-occurs with 17 other knowledge elements, covering 46% of the network nodes. This means that nearly half of all knowledge elements in the network have direct co-occurrence relationships with bioinformatics, demonstrating its strongest direct influence. This result provides supporting evidence for the proposition that AI technologies empower pharmaceuticals through integration with domain-specific informatics. The pharmaceutical knowledge element cluster, representing traditional knowledge elements in the pharmaceutical industry, ranked second and third in direct influence. The biotechnology cluster, which provides technical support for drug manufacturing, ranked within the top ten. The computer technology cluster, encompassing AI technologies such as machine learning, reinforcement learning, and neural networks, also ranked within the top ten, reflecting AI’s active pursuit of collaborative development opportunities in the pharmaceutical industry.

**Table 5:** Top 10 knowledge elements by influence.

Rank	Degree Centrality	PageRank
1	G16B (Bioinformatics)	G16B (Bioinformatics)
2	A61K (Pharmaceuticals preparations)	A61K (Pharmaceuticals preparations)
3	A61P (Therapeutic activity)	A61P (Therapeutic activity)
4	C12N (Biotechnology)	C12N (Biotechnology)
5	C07K (Biotechnology)	G01N (Biomaterial analysis)
6	G01N (Biomaterial analysis)	C07K (Biotechnology)
7	G06F (Computer technology)	G06F (Computer technology)
8	G16C (Cheminformatics)	G16C (Cheminformatics)
9	G06N (Computer technology)	G06N (Computer technology)
10	C12R (Biotechnology)	C12R (Biotechnology)

(2) **Identification of knowledge element brokerage capacity.** Betweenness centrality measures the frequency with which a node appears on the shortest paths between all possible pairs of nodes in the network. In knowledge element co-occurrence network analysis, betweenness centrality measures a knowledge element"s capacity to serve as a bridge connecting different knowledge domains. Knowledge elements with high betweenness centrality typically occupy critical connecting positions between different knowledge domains, functioning as knowledge hubs, whilst those with low betweenness centrality tend to be positioned at the network periphery. Closeness centrality measures the average distance from a given starting node to all other nodes. In knowledge element co-occurrence network analysis, knowledge elements with high closeness centrality can rapidly and directly establish connections with other knowledge domains, whereas those with low closeness centrality require multiple intermediate steps to connect with other knowledge domains.

As shown in Table 6, examining betweenness centrality and closeness centrality together, the **first tier** comprises G16B (bioinformatics), which encompasses information and communication technologies specially adapted for genetic or protein-related data processing, including nucleic acid folding, protein folding, and drug targeting using structural data. G16B (bioinformatics) simultaneously possesses the strongest bridging capacity and shortest path length in the co-occurrence network, constituting the core knowledge element in AI-driven drug discovery. The **second tier** consists of A61K and A61P (pharmaceuticals), primarily involving application-oriented knowledge in the pharmaceutical industry that connects theoretical research with actual products. The **third tier** includes C12N (biotechnology), C07K (biotechnology), and G01N (biomaterial analysis); these knowledge elements constitute foundational technologies for AI-driven drug discovery, providing technical support for translating drugs from concept to reality. The **fourth tier** comprises technology clusters centred on computer technology, including G06F, G06N, and G06V, indicating that computer technologies such as AI are not “optional tools” for the pharmaceutical industry but rather essential foundational technologies deeply embedded in the pharmaceutical industry’s technological network.

**Table 6:** Top 10 knowledge elements by brokerage capacity.

Rank	Betweenness Centrality	Closeness Centrality
1	G16B (Bioinformatics)	G16B (Bioinformatics)
2	A61K (Pharmaceuticals preparations)	A61K (Pharmaceuticals preparations)
3	G01N (Biomaterial analysis)	C12N (Biotechnology)
4	C12N (Biotechnology)	A61P (Therapeutic activity)
5	A61P (Therapeutic activity)	C07K (Biotechnology)
6	G16C (Cheminformatics)	G01N (Biomaterial analysis)
7	C07K (Biotechnology)	C12Q (Biotechnology)
8	G06F (Computer technology)	C12R (Biotechnology)
9	G06V (Computer technology)	G06F (Computer technology)
10	B82Y (Microstructure and nanotechnology)	G06N (Computer technology)

### LDA topic modelling

While IPC co-occurrence network analysis effectively captures “co-occurrence associations” between knowledge elements, it is limited in uncovering the underlying semantic logic within the “content dimension”, specifically regarding core technical objectives and application scenarios. To address this limitation, Latent Dirichlet Allocation (LDA) topic modelling was employed to mine semantic information from patent texts (titles and abstracts). Consequently, LDA serves not as a replacement, but as a complementary method to enhance the analysis. Specifically, clustering results from granted patent texts reflect the aggregation patterns of knowledge elements and technological focus within the field of AI-driven drug discovery over specific periods. Drawing on established methodologies^24^, this study extracted titles and abstracts from granted invention patents spanning 2011–2024 and applied LDA topic modelling for cluster analysis. As a generative probabilistic model, LDA assumes that documents consist of multiple topics, each comprising specific feature words, allowing for the inference of latent topics and associated keywords. To ensure reliability and reproducibility, the data processing workflow for the LDA model was implemented as shown in Table 7.

**Table 7:** Data processing procedures for the LDA topic classification model.

Step	Specific Operations
Data preprocessing	Cleaned and preprocessed titles and abstracts from 494 patents, including removal of stopwords, punctuation, and numbers, as well as stemming or lemmatisation
Topic number selection	Evaluated candidate topic numbers (K = 2–20) using multiple complementary metrics: C_V coherence, log perplexity, U_Mass coherence, silhouette coefficient, within-topic focus, and inter-topic similarity; the optimal K was determined through comprehensive assessment combined with domain-informed interpretability
Model training	Trained the model using the LDA algorithm with preprocessed data; the algorithm learns the topic distribution for each document and the word distribution for each topic through iterative estimation
Result interpretation	Examined each topic and its associated keywords to interpret topic meaning, with particular attention to AI-related thematic differentiation

To determine the optimal number of topics (K), we adopted a multi-metric evaluation framework rather than relying on a single indicator. Six complementary metrics were computed for K = 2 to 20: (1) C_V coherence score, which quantifies the semantic connectivity of keywords within a topic, with higher values indicating more interpretable topics; (2) log perplexity, a measure of model fit where lower values indicate better generalisation; (3) U_Mass coherence, an alternative coherence measure based on document co-occurrence where values closer to zero indicate better performance; (4) silhouette coefficient, which measures how well documents are assigned to their dominant topic versus other topics, with higher values indicating clearer topic separation; (5) within-topic focus, defined as the average probability of the top-10 keywords within each topic, with higher values indicating more concentrated and distinctive topics; and (6) inter-topic similarity, computed as the average pairwise cosine similarity between topic-word distributions, with lower values indicating greater differentiation among topics.

Table 8 presents the complete evaluation results. Several key patterns emerge from the multi-metric assessment. First, C_V coherence—the primary indicator of topic interpretability—reached its global maximum at K = 8 (0.4949), after which values fluctuated without systematic improvement, indicating that further topic subdivision does not yield meaningful gains in semantic coherence. Second, the silhouette coefficient at K = 8 (0.585) remained at an acceptable level, indicating adequate document-topic assignment quality. Third, inter-topic similarity at K = 8 (0.216) was substantially lower than at smaller K values, demonstrating that the eight-topic solution produces well-differentiated thematic clusters with limited redundancy among topics. Fourth, within-topic focus at K = 8 (0.280) showed meaningful improvement over lower K values, indicating that each topic is internally concentrated around a distinctive set of keywords. Based on these comprehensive quantitative assessments, combined with expert evaluation of topic interpretability—particularly the capacity to distinguish among AI-related research themes—K = 8 was selected as the optimal number of topics..

**Table 8:** Multi-metric evaluation results for topic number selection (K = 2–20).

K	Log Perplexity (lower is better)	C_V Coherence (higher is better)	U_Mass Coherence (closer to 0 is better)	Silhouette Coefficient (higher is better)	Within-Topic Focus (higher is better)	Inter-Topic Similarity (lower is better)
2	− 5.8425	0.3391	− 5.6195	0.83	0.178	0.548
3	− 5.7352	0.4226	− 4.5548	0.7483	0.2041	0.4091
4	− 5.7061	0.4093	− 5.4768	0.6491	0.2186	0.3513
5	− 5.6256	0.4341	− 6.3090	0.6473	0.2339	0.2745
6	− 5.5888	0.4625	− 5.1334	0.6222	0.2487	0.2571
7	− 5.5572	0.4629	− 6.6711	0.5906	0.2651	0.2407
8	− 5.5254	0.4949	− 6.2687	0.5852	0.2799	0.2155
9	− 5.4967	0.4833	− 6.3285	0.5765	0.2973	0.2017
10	− 5.4893	0.4532	− 7.2277	0.5757	0.295	0.2088
11	− 5.4396	0.4242	− 6.9961	0.6041	0.3095	0.1874
12	− 5.4199	0.4453	− 6.6028	0.5803	0.3208	0.1767
13	− 5.4227	0.4889	− 6.0101	0.5923	0.3267	0.1786
14	− 5.4040	0.487	− 6.0918	0.5809	0.3387	0.1626
15	− 5.4191	0.4754	− 6.4503	0.5711	0.3399	0.1796
16	− 5.3446	0.4643	− 6.5075	0.592	0.3461	0.1333
17	− 5.3604	0.4646	− 5.9613	0.5827	0.3558	0.1397
18	− 5.3691	0.4438	− 6.2652	0.5575	0.3745	0.1263
19	− 5.3455	0.4726	− 5.5196	0.5687	0.3712	0.1286
20	− 5.3595	0.4726	− 5.7350	0.5504	0.3734	0.1332

Note: K = 8 (bold) achieves the highest C_V coherence score (0.4949) while maintaining acceptable levels across all other metrics.

The specific clustering results for each topic are presented in Table 9. The eight topics collectively reveal a rich and well-differentiated thematic landscape of AI-driven drug discovery, particularly in distinguishing among AI-related and computational research themes.

**Table 9:** Clustering results for patent texts (K = 8).

No.	Topic Label	Keywords
1	Cell Biology and Crystal-Based Drug Formulation	cell, crystal, IL-17, interleukin, induction, drug, stem cell, serum, neural, treatment
2	Compound Screening and Ligand-Based Drug Design	compound, drug, chemical reaction, ligand, combination, DNA, display, preparation, alcoholic, diabetes
3	Pharmaceutical Compounds and Therapeutic Applications	treatment, compound, drug, preparation, combination, administration, inhibitor, pharmaceutical, prevention, activity
4	Molecular Force Field Parameterisation and Structural Analysis	molecule, force field, parameter, fragment, polypeptide, computation, analysis, atom, mutation, fitting
5	Computational Infrastructure and Polymorph Prediction	computation, crystal form, service, molecule, free energy, user, energy, execution, job, management
6	AI-Driven Molecular Generation and Protein Structure Design	molecule, sequence, structure, protein, computation, generation, multiple, candidate, conformation, acquisition
7	Machine Learning Prediction Models and Biomedical Data Analytics	model, prediction, data, gene, training, based on, molecule, sample, tumour, cell
8	Biological Sequence Analysis and Immunological Detection	sequence, protein, molecule, detection, mRNA, biology, sample, immune, response, mutation

Topic 1: Cell Biology and Crystal-Based Drug Formulation. This topic centres on biological experiments and pharmaceutical formulation research, with keywords such as “cell”, “crystal”, “IL-17”, “stem cell”, “serum”, and “neural” reflecting work on cell-based assays, cytokine-related therapies, and crystal engineering for drug delivery systems. It captures the biological foundation upon which AI-driven drug discovery ultimately rests—the experimental validation and formulation stages that remain essential even as computational methods advance.

Topic 2: Compound Screening and Ligand-Based Drug Design. Keywords including “compound”, “chemical reaction”, “ligand”, “DNA”, and “screening” characterise this topic as focused on the identification and optimisation of drug candidates through ligand-receptor interaction analysis and compound library screening. The presence of disease-specific terms such as “alcoholic” and “diabetes” indicates that these screening efforts target specific therapeutic areas. This topic represents the interface where computational predictions meet chemical reality through reaction design and ligand evaluation.

Topic 3: Pharmaceutical Compounds and Therapeutic Applications. Dominated by keywords such as “treatment”, “compound”, “drug”, “preparation”, “inhibitor”, and “pharmaceutical”, this topic captures the core of traditional pharmaceutical R&D—the development of therapeutic agents from compound synthesis through formulation to clinical application. The high probability weights of these keywords (treatment: 0.0556; compound: 0.0504; drug: 0.0480) confirm that conventional pharmaceutical knowledge remains the foundational anchor of AI-driven drug discovery, consistent with the IPC co-occurrence finding that A61K–A61P constitutes the highest-intensity knowledge combination.

Topic 4: Molecular Force Field Parameterisation and Structural Analysis. This topic is characterised by computational chemistry terminology: “force field”, “parameter”, “fragment”, “atom”, “fitting”, and “polypeptide”. It represents the development and refinement of molecular mechanics force fields—mathematical models that approximate the potential energy of molecular systems. Force field parameterisation is a prerequisite for molecular dynamics simulations and plays a foundational role in structure-based drug design, where accurate energy calculations determine the reliability of binding affinity predictions.

Topic 5: Computational Infrastructure and Polymorph Prediction. Keywords such as “computation”, “service”, “execution”, “job”, “management”, and “user” alongside “crystal form” and “free energy” reveal an infrastructure-oriented topic that captures patents related to high-performance computing platforms and cloud-based services designed for computationally intensive pharmaceutical tasks such as polymorph screening and free energy calculations. This topic reflects the growing importance of computational infrastructure as an enabling layer for AI-driven drug discovery—the deployment of scalable computing services that allow researchers to execute complex simulations and predictions at scale.

Topic 6: AI-Driven Molecular Generation and Protein Structure Design. This is the first of two explicitly AI-centred topics. The dominant keyword “molecule” (probability: 0.1072) co-occurs with “sequence”, “structure”, “protein”, “generation”, “candidate”, and “conformation”, indicating a focus on generative AI approaches to molecular and protein design. This topic captures patents employing deep generative models—such as variational autoencoders, generative adversarial networks, and diffusion models—to produce novel molecular candidates with desired structural and functional properties. The emphasis on “generation” and “candidate” distinguishes this topic from purely analytical approaches, highlighting the creative dimension of AI in drug discovery: the capacity to design molecules that do not yet exist in known chemical libraries. Representative patents within this topic illustrate specific generative AI approaches currently deployed in drug discovery. For instance, one patent (CN117809749B, PureRidge Biotech/Chinese Academy of Sciences, 2024) employs a pre-trained protein language model combined with a diffusion-based generative model to design de novo functional polypeptide sequences without known templates or predetermined target structures—exemplifying the template-free generative capacity that distinguishes this paradigm. Another patent (CN118866165B, DP Technology, 2024) constructs a generative model specifically for polycyclic conjugated systems, enabling both de novo molecular generation and structure optimisation with confidence scoring. A third patent (CN113096723B, XtalPi, 2021) integrates a molecular generation module with transfer learning to produce bioactive drug-like small molecules targeting specific receptors, combining generative design with activity prediction to construct target-directed molecular libraries. These examples demonstrate that the generative paradigm in AI-driven drug discovery encompasses a range of AI architectures—including protein language models, diffusion models, and transfer learning frameworks—applied to tasks spanning peptide design, small-molecule generation, and structure optimisation.

Topic 7: Machine Learning Prediction Models and Biomedical Data Analytics. This is the second AI-centred topic. Keywords including “model”, “prediction”, “data”, “training”, and “based on” clearly indicate machine learning methodologies, while “gene”, “sample”, “tumour”, and “cell” anchor these methods in biomedical applications. This topic represents the data-driven predictive paradigm in AI-driven drug discovery, encompassing patents that employ supervised and unsupervised learning algorithms—such as neural networks, random forests, and support vector machines—to predict molecular properties, identify biomarkers, forecast drug-target interactions, and analyse genomic and transcriptomic data for therapeutic target identification. Representative patents within this topic illustrate the diversity of predictive AI models applied to pharmaceutical tasks. One patent (CN118398125B, MoleculeAI/Jitai Pharma, 2024) fine-tunes a pre-trained BERT model on lipid molecular data to predict molecular properties, demonstrating the adaptation of natural language processing architectures to chemical representation learning. Another patent (CN112201313B, XtalPi, 2020) trains a predictive model on molecular structural features and activity data to screen candidate molecules with high predicted activity against a specified target, representing a classic supervised learning pipeline for virtual screening. A third patent (CN112185458B, XtalPi, 2020) employs convolutional neural networks to predict protein–ligand binding free energy from three-dimensional structural representations, enabling rapid and accurate estimation of binding affinity without computationally expensive physics-based simulations. These examples illustrate that the predictive paradigm spans multiple AI architectures—including transformer-based language models (BERT), supervised learning pipelines, and convolutional neural networks—applied across the drug discovery pipeline from molecular property prediction through virtual screening to binding affinity estimation.

Topic 8: Biological Sequence Analysis and Immunological Detection. Keywords including “sequence”, “protein”, “mRNA”, “detection”, “immune”, “response”, and “mutation” characterise this topic as focused on biological sequence analysis and immunological applications. It captures patents involving protein and nucleic acid sequence analysis, mRNA-based therapeutic design, immune response profiling, and mutation detection—activities that provide the biological data inputs upon which AI prediction models (Topic 7) and generative design approaches (Topic 6) operate.

The eight-topic structure offers three notable analytical insights. First, it differentiates two distinct AI-related themes—generative molecular design (Topic 6) and predictive data analytics (Topic 7)—rather than treating AI as a single undifferentiated category.The generative paradigm (Topic 6) aims to create novel molecular entities, whereas the predictive paradigm (Topic 7) aims to evaluate existing entities or datasets—a distinction analogous to the generative–discriminative divide in machine learning.This differentiation reveals that AI is not a monolithic force in drug discovery but operates through at least two distinct technological paradigms: a generative paradigm focused on creating novel molecular candidates, and a predictive paradigm focused on learning patterns from existing biomedical data. Second, the topic structure identifies the computational infrastructure layer (Topic 5) as a distinct thematic domain, highlighting the engineering dimension of AI-driven drug discovery that might otherwise be obscured at coarser levels of thematic aggregation. Third, the traditional pharmaceutical domain is decomposed into nuanced sub-themes—cell biology and formulation (Topic 1), compound screening (Topic 2), and therapeutic applications (Topic 3)—providing a detailed view of how conventional pharmaceutical knowledge is organised within AI-driven drug discovery patents.

## Evolutionary analysis

To address Research Question 2, this section adopts a dynamic perspective by employing a sliding window network analysis. Continuous knowledge element co-occurrence networks were constructed using a 5-year window with a 1-year step size to trace the evolutionary trajectories of knowledge recombination within the field of AI-driven drug discovery. The analysis proceeds in two sequential stages: Evolution of network scale and structure Section characterizes the evolution of the network’s scale and topological structure based on dynamic co-occurrence networks; Evolutionary trajectories of core knowledge elements Section delineates the evolutionary trajectories of core knowledge elements, focusing on key nodes identified by degree centrality and betweenness centrality.

### Evolution of network scale and structure

**Fig. 5:**
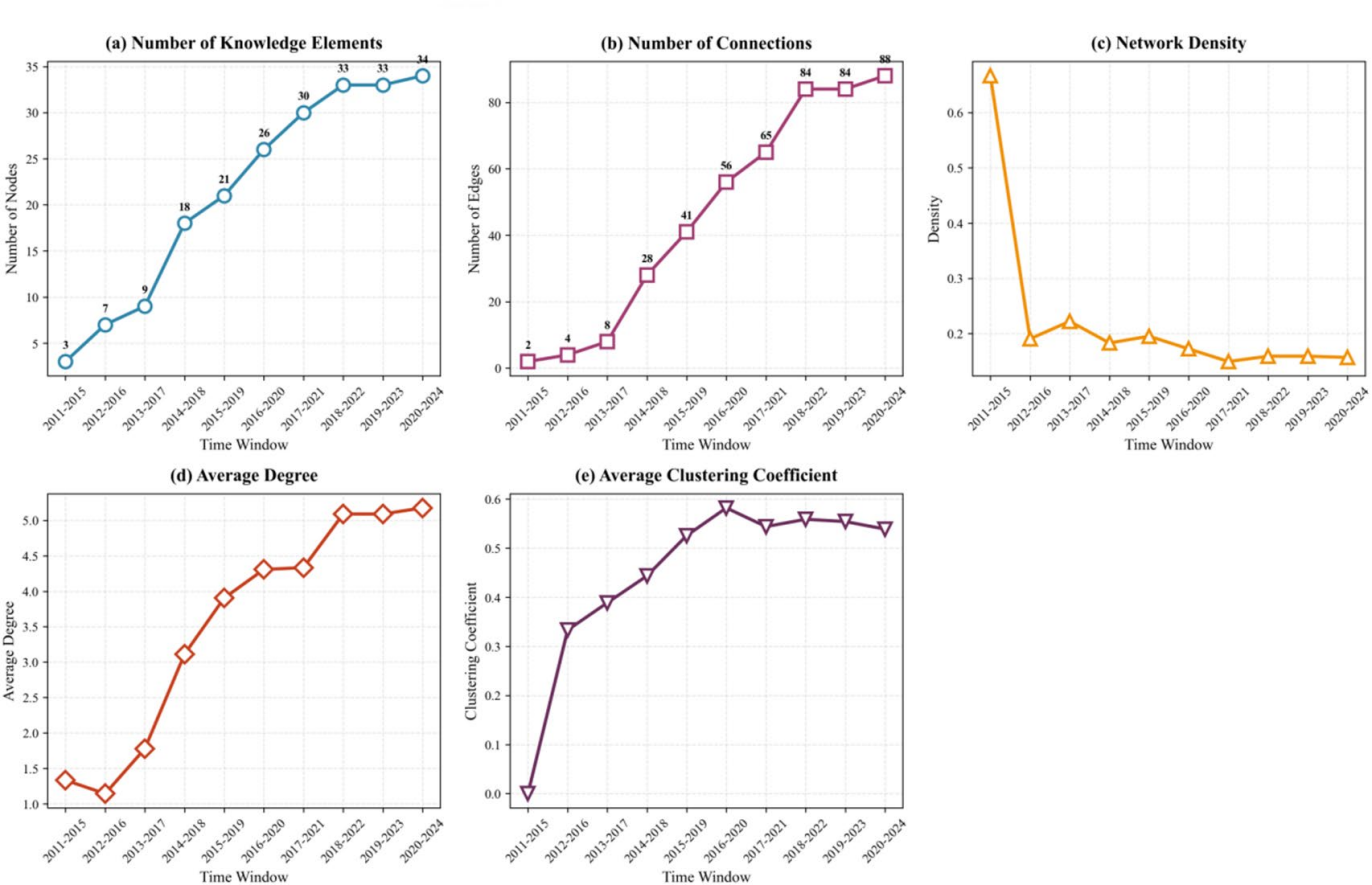
Evolution of network-level indicators across sliding windows.

**Fig. 6:**
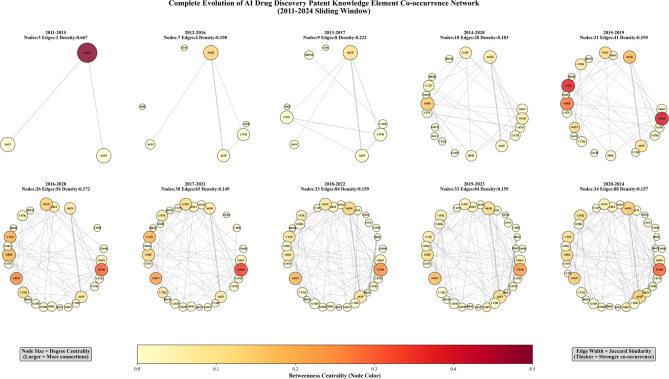
Network topology visualisations for sliding windows.

Based on the Jaccard-weighted co-occurrence matrices constructed for each sliding window, we computed five network-level indicators to characterise the evolution of network scale and structure. Table 10 summarises the definition and interpretation of each indicator. Figure 5 presents the evolution of these indicators across the ten sliding windows, and Fig. 6 visualises the corresponding network topologies.

**Table 10:** Network-level indicators and their interpretation.

Indicator	Definition	Interpretation
Node count	Number of distinct IPC subclasses appearing in the window	Technological diversity of the knowledge network
Edge count	Number of co-occurrence relationships between IPC subclasses	Volume of cross-domain technological associations
Network density	Ratio of actual edges to the maximum possible edges	Overall connectivity; higher values indicate more complete integration among elements
Average degree	Mean number of connections per node	Breadth of association for a typical knowledge element
Clustering coefficient	Ratio of actual to possible connections among a node’s neighbours	Local clustering tendency; higher values indicate formation of tightly connected technology clusters

Two findings emerged from the analysis:

First, network scale expanded substantially over the observation period. Node count increased from 3 (2011–2015) to 34 (2020–2024), and edge count increased from 2 to 88, reflecting the continuous entry of heterogeneous technological elements into the AI-driven drug discovery domain.

Second, a **temporal lag** characterizes the process of knowledge integration. As observed, network density declined from 0.667 to 0.157, whereas the average degree simultaneously increased from 1.33 to 5.18. This divergence reveals a lag in integration: the influx of newly entering elements initially dilutes overall network connectivity, while core elements simultaneously strengthen and expand their existing connections. This phenomenon is **epitomized** by the evolutionary trajectory of **Bioinformatics (G16B)**, which transitioned from **absence to emergence**,** and ultimately to a status as a core hub**. During the initial window (2011–2015), G16B was not positioned within the core network structure. However, as technological convergence deepened, G16B rapidly ascended to become the node with the highest degree centrality during the 2017–2021 window, sustaining this dominant position in all subsequent periods.

Third, the clustering coefficient exhibited a pattern of initial rapid increase followed by convergence. During the early windows (2011–2015 to 2016–2020), the clustering coefficient rose sharply from 0.000 to a peak of approximately 0.58, reflecting the rapid formation of tightly connected technology clusters—such as the pharmaceutical chemistry cluster (A61K–A61P–C07D) and the AI-informatics cluster (G06N–G16B–G16C)—in which core knowledge elements quickly established closed triadic relationships with one another. However, from the 2017–2021 window onward, the clustering coefficient stabilised within a narrow range of approximately 0.54–0.56, despite continued growth in node count and average degree. This convergence suggests that the internal structure of core technology clusters had approached saturation by this stage: the triadic relationships among established core nodes were largely complete. Subsequently entering knowledge elements tended to connect to established core nodes without forming closed triads with the existing neighbours of those nodes, thereby contributing to network expansion (rising node count and average degree) without further elevating local clustering. This pattern complements the temporal lag phenomenon described above: while the declining density and rising average degree reveal the macro-level dynamics of lagged integration, the convergence of the clustering coefficient reveals the micro-structural mechanism underlying this process—new elements are initially attached to core hubs rather than embedded into existing tightly knit sub-groups.

### Evolutionary trajectories of core knowledge elements

**Fig. 7:**
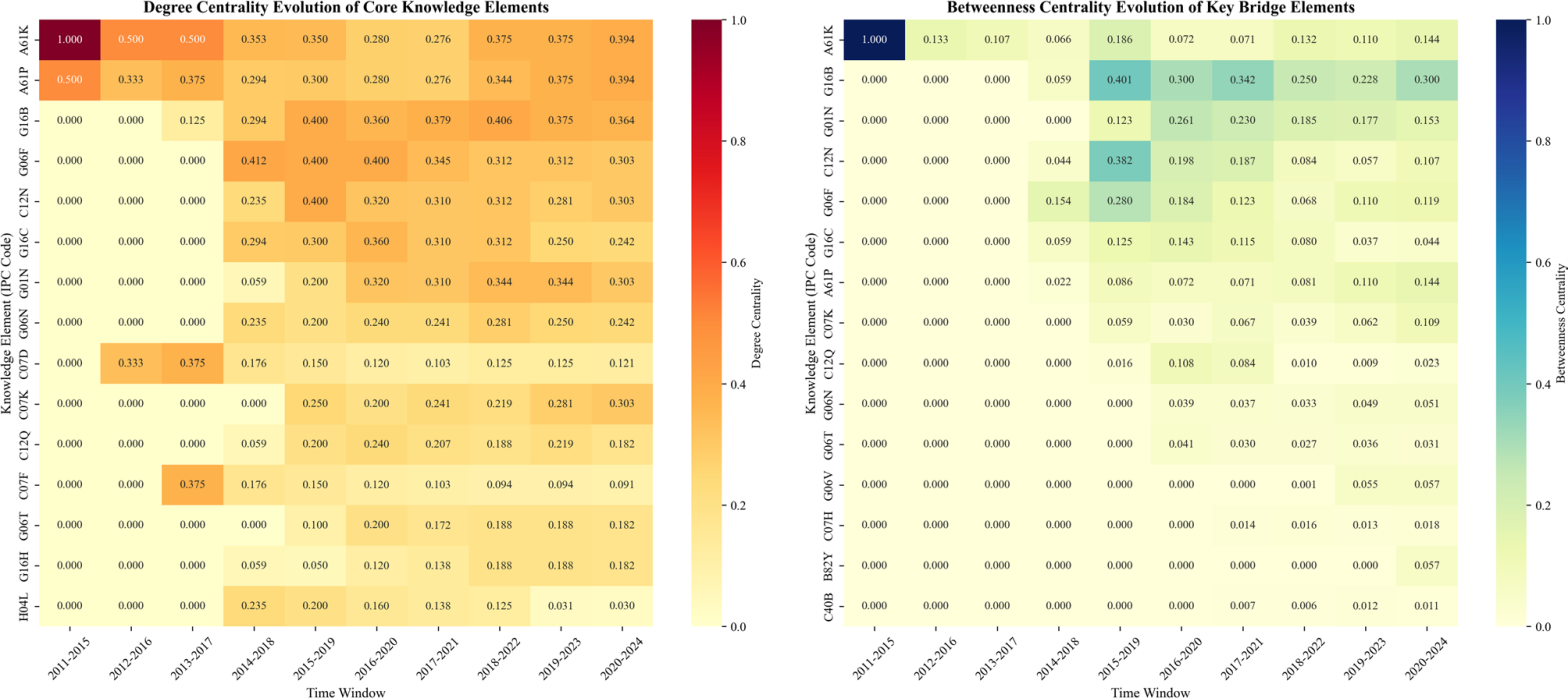
Evolutionary heatmap of core knowledge elements.

Centrality analysis based on sliding windows further elucidates the micro-level succession trajectories of core technical elements, which are primarily manifested in two dimensions:

First, the evolution of core nodes based on degree centrality demonstrates a shift in technological dominance. As illustrated in Fig. 7, during the 2011–2015 period, the network was dominated by the traditional pharmaceutical element A61K (Preparations for medical purposes), which exhibited a degree centrality of 1.000. In the 2014–2018 window, G06F (Electric digital data processing) emerged prominently, rising to the top position with a centrality of 0.412. This marks the robust intervention of external computing technologies. Subsequently, from 2015 to 2024, G16B (Bioinformatics) gradually superseded single-technology nodes. It reached peak centrality (0.406) during the 2018–2022 window. By the 2019–2024 period, both G16B and A61K jointly occupied the leading positions in centrality. This evolutionary path—transitioning from “Pharmaceuticals” to “Computing”, then to “Bioinformatics” ,and finally to “Multidisciplinary Dominance”—reflects the deepening of technological convergence and fusion within the AI-driven drug discovery field.

Second, the evolution of bridge nodes based on betweenness centrality reveals a restructuring of the “connection mechanism”. As shown in Fig. 6, the early-stage network lacked effective intermediary nodes, relying almost exclusively on A61K to maintain internal connectivity. As the evolution progressed, G16B (Bioinformatics) demonstrated the highest betweenness centrality (0.401) during the 2015–2019 window, consistently serving as a critical hub bridging “computing technology” and “biomedicine”.

## Discussion and conclusions

### Conclusion

This study analysed 494 granted invention patents from 58 Chinese AI pharmaceutical firms spanning 2011–2024, providing a systematic empirical characterisation of knowledge recombination in AI-driven drug discovery.

Addressing RQ1 (structural characteristics), four key findings emerged. First, knowledge recombination exhibits a “sparse yet concentrated” network architecture: only 14.71% of possible knowledge element pairs co-occur, yet co-occurrence frequencies follow a long-tail distribution with a few core combinations dominating. Second, bioinformatics (G16B) serves as the central bridging hub, displaying the highest degree centrality (connecting 46% of network nodes), betweenness centrality, and closeness centrality—a unique structural position attributable to its dual computational-biomedical nature. Third, comparison between combinatorial intensity (Jaccard coefficient) and semantic similarity (cosine similarity) reveals that AI-driven combinations exhibit high combinatorial intensity despite low semantic similarity, providing evidence for distant recombination across cognitive boundaries. Fourth, LDA topic modelling—informed by a multi-metric evaluation framework encompassing six complementary indicators across K = 2 to 20—identifies eight thematic domains: cell biology and crystal-based drug formulation, compound screening and ligand-based drug design, pharmaceutical compounds and therapeutic applications, molecular force field parameterisation, computational infrastructure and polymorph prediction, AI-driven molecular generation and protein structure design, machine learning prediction models and biomedical data analytics, and biological sequence analysis and immunological detection. The topic structure suggests the presence of two distinct AI-related paradigms within the corpus: a generative paradigm oriented towards novel molecular candidate creation (Topic 6) and a predictive paradigm oriented towards data-driven pattern recognition (Topic 7).

Addressing RQ2 (evolutionary trajectory), three patterns were documented. First, network scale expanded substantially: node count increased from 3 to 34, and edge count from 2 to 88. Second, network density declined (from 0.667 to 0.157) concurrent with rising average degree (from 1.33 to 5.18), indicating continuous influx of new technologies alongside strengthening integration among core elements—a pattern reflecting temporal lag in knowledge integration. Third, bioinformatics exemplifies this trajectory, evolving from absence in early windows to the dominant hub in later periods.

### Discussion

This study provides a systematic empirical characterisation of knowledge recombination in AI-driven drug discovery through multi-method patent analysis. The findings contribute to the existing literature in five respects, which we discuss in turn.

**Knowledge recombination in AI-driven drug discovery exhibits a “sparse yet concentrated” structure.** Our analysis indicates that only 14.71% of the 666 theoretically possible knowledge element pairs actually co-occur, yet co-occurrence frequencies are highly uneven, with the mean (7.11) approximately 3.56 times the median (2), exhibiting a pronounced long-tail pattern. This finding resonates with the macro-level observation of previous studies, which documented power-law distributions in technological combinations across millions of US patents^18^. However, whereas their analysis aggregated across the entire patent database, our study focuses specifically on AI-driven drug discovery and identifies the particular knowledge combinations around which recombination activity concentrates. The highest-intensity combinations include traditional pharmaceutical pairings (A61K-A61P, Jaccard = 0.90), AI-bioinformatics integrations (G06N-G16B, Jaccard = 0.20), and AI-cheminformatics integrations (G06N-G16C, Jaccard = 0.18). These core combinations appear to constitute the technological anchors of the field, suggesting that cross-domain recombination may not proceed through exhaustive exploration of the combinatorial space but instead concentrates on a selective subset of productive pairings.

**Bioinformatics serves as a bridging hub between distant knowledge domains.** Network centrality analysis identifies bioinformatics (G16B) as the most structurally prominent knowledge element, ranking first simultaneously in degree centrality (connecting 46% of network nodes), betweenness centrality, and closeness centrality (Tables 4 and 5). This finding directly addresses the bridging mechanism question raised by the cognitive distance framework of previous studies, which established that knowledge integration becomes increasingly difficult as cognitive distance between domains grows^9^, yet without specifying what structural mechanisms might bridge such gaps. Similarly, Cohen and Levinthal proposed that “related prior knowledge” facilitates the absorption of external knowledge^36^, but did not define what such intermediate knowledge looks like in cross-domain contexts. Our results suggest that bioinformatics occupies this bridging role due to its dual computational-biomedical nature: it possesses data processing and algorithmic attributes compatible with AI technologies (G06 series), while maintaining deep roots in biological and medical applications (A61 and C12 series). The comparison between combinatorial intensity and semantic similarity reinforces this interpretation. AI-bioinformatics combinations (e.g., G06N-G16B) exhibit high combinatorial intensity (Jaccard = 0.20) despite low semantic similarity (cosine = 0.3775), whereas traditional pharmaceutical combinations (e.g., A61K-C12N) show the reverse pattern (Jaccard = 0.13, cosine = 0.7888). This contrast is consistent with the interpretation that bioinformatics may enable genuine distant recombination across cognitive boundaries, rather than merely facilitating combination among semantically proximate domains. At the firm level, Hargadon and Sutton documented technology brokering as a mechanism for bridging multiple domains^37^. Our findings may extend this concept from the organisational level to the knowledge structure level, pointing to a specific knowledge-level characteristic—dual-domain attributes—that enables bridging functions within co-occurrence networks.

**Knowledge integration exhibits temporal lag during network evolution.** The sliding-window analysis documents a notable divergence: network density declined from 0.667 to 0.157 whilst average degree simultaneously increased from 1.33 to 5.18 over the observation period. This pattern indicates that newly entering knowledge elements do not immediately establish extensive connections with existing elements; instead, integration proceeds gradually as core nodes consolidate their positions. This finding is consistent with the proposition of previous studies, which established that general-purpose technologies (GPTs) generate value through progressive integration with application domains, rather than through abrupt substitution^38^. The concurrent rise in clustering coefficient (from 0.000 to approximately 0.55) further suggests that as the field matures, localised technology clusters form around core nodes, indicating deepening integration within specific technological neighbourhoods. Drawing on the knowledge-based view and organizational learning literature, three complementary mechanisms may account for this lag. First, absorptive capacity constraints^36^ suggest that the assimilation of new knowledge elements requires a stock of related prior knowledge that accumulates only over time—pharmaceutical nodes must develop computational familiarity, and AI nodes must acquire biomedical understanding, before productive connections can form. Second, cognitive coordination costs⁹ imply that bridging cognitively distant domains demands costly translation and reconciliation efforts; the low semantic similarity between AI and pharmaceutical knowledge elements documented in Jaccard similarity matrix and combinatorial intensity–cognitive distance comparison Section (e.g., G06N–G16B, cosine = 0.3775) offers supporting evidence for such distance. Third, the exploration–exploitation framework^39^ offers a broader lens: the declining density corresponds to an exploration phase in which the combinatorial space expands, while the rising average degree corresponds to an exploitation phase in which core nodes consolidate around validated combinations. Together, these mechanisms suggest that in cross-domain convergence, network scale expansion tends to precede connectivity consolidation, with the temporal gap shaped by absorptive capacity accumulation and cognitive coordination costs.This proposition, however, remains a hypothesis derived from descriptive network patterns and would benefit from further verification through longitudinal studies with larger and more diverse patent corpora.

**Core knowledge elements exhibit dynamic evolutionary trajectories.** Previous studies suggested that knowledge recombination evolves dynamically^27^, but the specific temporal patterns of such dynamics in cross-domain convergence had not been empirically documented. Our data provide descriptive evidence for these dynamics: the evolution of core node dominance traces a clear trajectory—from traditional pharmaceutical elements (A61K) in the 2011–2015 window, through computer technology (G06F) in the 2014–2018 window, to bioinformatics (G16B) from the 2017–2021 window onward (Fig. 6). This shift in hub positions suggests that AI has progressively transitioned from an external computational tool to a deeply embedded component of pharmaceutical innovation, mediated by the rise of bioinformatics as the integrative bridge. We note, however, that our descriptive approach does not permit causal inference regarding the mechanisms driving this trajectory.

**The eight-topic structure suggests that AI’s role in drug discovery is not monolithic.** The differentiation between generative molecular design (Topic 6) and predictive data analytics (Topic 7) suggests that AI-driven drug discovery involves at least two distinct knowledge integration mechanisms: one centred on the creation of novel chemical entities through generative models, and another centred on the extraction of predictive patterns from biomedical datasets. This thematic differentiation complements the structural finding from IPC co-occurrence analysis that bioinformatics (G16B) serves as the bridging hub: while the network analysis identifies which knowledge domains combine, the topic modelling reveals the distinct functional objectives underlying these combinations.Whether this bifurcation reflects a fundamental structural feature of AI-driven drug discovery or is specific to the present corpus warrants further investigation with expanded datasets.

### Implications

The findings of this study offer practical insights for three groups of stakeholders.

**Implications for AI pharmaceutical firms.** First, the “sparse yet concentrated” structure of knowledge recombination suggests that firms may benefit from prioritising resource allocation towards established high-intensity combinations rather than dispersing efforts across the entire combinatorial space. Second, the central hub position of bioinformatics (G16B) in the co-occurrence network points to its potential role as an integrative bridge between AI technologies and pharmaceutical sciences. Firms operating in this field may benefit from attending to advances in bioinformatics and related domain-specific informatics, seeking productive intersections with AI methods to strengthen their cross-domain integration capabilities. Third, the temporal lag in knowledge integration documented by the sliding-window analysis indicates that newly introduced technologies require time to establish connections within the existing knowledge network. Firms may therefore need to maintain realistic expectations regarding the integration timeline of novel technological elements and allow sufficient time for new capabilities to be absorbed into their innovation processes.

**Implications for policymakers.** The identification of bioinformatics as a bridging domain suggests that targeted support for “intermediate knowledge domains”—fields possessing dual attributes that span distant disciplinary boundaries—may be particularly effective in facilitating cross-domain technological convergence. Policymakers could consider dedicated funding and platform-building initiatives for bioinformatics and comparable bridging disciplines (e.g., cheminformatics, computational biology) to accelerate AI-pharmaceutical integration. Furthermore, the evolutionary trajectory documented in this study—characterised by expanding network scale, shifting hub positions, and temporal lag in knowledge integration—suggests that support strategies could be calibrated to the maturation stage of the field.

**Implications for educational institutions.** The central bridging role of bioinformatics in connecting AI technologies and pharmaceutical sciences underscores the potential value of interdisciplinary curricula designed to cultivate hybrid expertise spanning computational methods and biomedical sciences. More broadly, fostering the development of cross-disciplinary fields at the intersection of AI and other domains—such as domain-specific informatics—may lay the talent foundation for intelligent transformation across a wider range of sectors.

### Limitations and future research

Several limitations of this study should be acknowledged, each of which suggests directions for future research.

**First**,** the IPC-based co-occurrence approach captures certain dimensions of knowledge recombination while leaving others unobserved.** IPC co-occurrence analysis can reveal which knowledge domains participate in recombination, how frequently specific combinations occur, and what structural patterns characterise the overall combinatorial landscape. However, co-occurrence alone cannot reveal whether high-frequency combinations reflect cognitive proximity between domains or genuine distant recombination, nor can it uncover the R&D objectives and application contexts underlying these combinations. We addressed these two specific limitations through complementary methods: embedding-based cosine similarity analysis (using the BGE-large-zh-v1.5 model) was introduced to distinguish combinatorial intensity from cognitive distance, and LDA topic modelling was applied to uncover the thematic content underlying IPC-based structural patterns. Nonetheless, even with these additions, the analytical framework cannot capture the specific technical implementation details of how knowledge elements are integrated within individual patents. Future research could complement the present approach with fine-grained textual analysis of patent claims, which more precisely delineate the inventive contribution of each technological component.

**Second**,** this study operates at the IPC subclass level (first four digits)**,** which may limit the granularity of the analysis.** For a highly specialised field such as AI-driven drug discovery, analysis at the group or subgroup level could yield more fine-grained insights into the specific technological combinations at work. We adopted the subclass level for two reasons: (1) the relatively limited sample size (494 patents, 37 unique subclasses) means that finer-grained classification would produce an excessively sparse co-occurrence matrix, undermining the reliability of network analysis; and (2) established studies employing similar co-occurrence approaches have operated at comparable granularity levels^40^. A supplementary analysis at the six-digit (main group) level confirmed that finer-grained classification substantially increases matrix sparsity (fill rate: 8.80% vs. 14.71%) and network fragmentation (28.3% of nodes with degree ≤ 5), supporting the choice of the subclass level for the present sample size. The complete six-digit results are reported in Appendix C. Future research based on larger patent samples could explore group- or subgroup-level analysis to provide more detailed characterisation of knowledge recombination patterns.

**Third**,** this study focuses exclusively on patents from dedicated AI pharmaceutical firms**,** excluding patents from academic institutions**,** hospitals**,** and traditional pharmaceutical companies.** This scope restriction constitutes a significant limitation, as academic institutions have been significant contributors to early-stage AI-pharmaceutical research and may exhibit different recombination patterns—potentially more exploratory and diverse—than those observed in commercially oriented firms. We adopted this focus because dedicated AI pharmaceutical firms have been AI-centric since inception, providing a relatively clean sample for observing “native” cross-domain knowledge recombination without the confounding effects of legacy pharmaceutical patent portfolios. Nonetheless, the exclusion of these participants may introduce bias into the evolutionary trajectory presented. Future research should extend the sample to include patents from academic institutions and traditional pharmaceutical firms, enabling comparative analysis of knowledge recombination patterns across different types of innovators.

**Fourth**,** the patent data for the most recent years (2023–2024) are likely incomplete due to the time lag inherent in patent examination and publication.** Chinese invention patents typically require two to three years from application to grant, meaning that patents applied for in recent years may not yet appear in the granted patent database. This caveat applies particularly to the later sliding windows in the evolutionary analysis and technological innovation activities in 2023–2024 may have been more active than what is observed in the data. Future research should revisit the findings for this period once a more complete dataset becomes available.

**Fifth**,** this study focuses on the Chinese context**,** and the generalisability of the findings to other national or institutional settings requires further examination.** Different countries may exhibit distinct patterns of AI-pharmaceutical knowledge recombination due to variations in innovation ecosystems, intellectual property regimes, and industrial structures. Future research could extend the analytical scope to AI pharmaceutical firms across multiple countries, conducting cross-national comparative studies to assess whether the structural characteristics and evolutionary patterns identified here hold across different institutional environments.

**Sixth**,** this study employs a descriptive and correlational research design based on network analysis and topic modelling**,** which precludes causal inference.** While we have drawn on established theoretical frameworks—including absorptive capacity theory, cognitive coordination costs, and the exploration–exploitation perspective—to interpret the observed patterns, the relationships identified (e.g., the temporal lag phenomenon, the bridging role of bioinformatics) represent empirical associations rather than causally established mechanisms. Future research could employ longitudinal case studies, quasi-experimental designs, or process-tracing methodologies to examine the causal pathways through which cross-domain knowledge recombination unfolds in AI-driven drug discovery.

## Supplementary Information

Below is the link to the electronic supplementary material.

Appendix A-Symmetric raw co-occurrence frequency matrix

Appendix B-The complete Jaccard similarity matrix is provided

Appendix C-6-digit IPC symmetric raw co-occurrence frequency matrix

## Data Availability

The patent data analysed in this study were retrieved from the IncoPat patent database (https://www.incopat.com), a commercial database requiring subscription access. The list of AI pharmaceutical firms was derived from the AI pharmaceutical industry report released by Intellectual Medicine Bureau in 2023. The raw co-occurrence frequency matrix and Jaccard similarity matrix generated during this study are provided in Appendices A and B. Additional processed datasets are available from the corresponding author upon reasonable request.
